# Let Us Be up and Doing

**Published:** 1907-01

**Authors:** 


Let Us Be Up and Doing. Already a bill for a State Board of Examiners for Osteopathic physicians has been introduced in the Texas Senate by Willacy, and a bill for a State Board of Examination for Physio-medical physicians has been introduced in the

House by Lane, the first week of the session. A bill for an Osteopath Board is now before Congress for the District of Columbia.

Neither the State Medical Association bill for a Board of Medical Examiners nor the Anatomical Bill has yet been introduced at the present writing (January 15). Our legislative committee should lose no time in getting them in, for there will be the usual hard fight, and "our friends" have gotten the start. They will oppose anything that interrupts the present blissful state of repose they enjoy under the monstrosity called the "Medical Practice Act." It exempts altogether from examination those who give no drugs, as if that were the highest qualification and entitled them to special privileges. It gives them special privileges contrary to the Constitution.

The bill drafted by the State Medical Association and perfected by the House of Delegates at Fort Worth last April, is a fairly good one. I see no objection to it. All reference to "schools" was cut out at Fort Worth, and it simply provides for a Board of Medical Examiners composed of eleven physicians of character and standing, to be appointed by the Governor. The composition of the board is entirely at his discretion, without dictation or suggestion from any "school," and he may or may not build it entirely of "regular," "irregular," or "defective" (like our verbs) material; all regular, all osteopaths, all homo's, or half and half, or a sprinkle of each, as he sees fit. It provides for examination only on branches common to all "schools," and from our standpoint it should be satisfactory to all honest practitioners of whatever "school" who want to do right, and do not fear examination. Should the Governor give what we call the "irregulars" a predominating influence on the board, our graduates would have to be examined, and there would be no objection. I'm sure they would not kick.

The bill is perhaps the best that can be gotten up, with any likelihood whatever of passing; as the former efforts to compel examination of the drugless element have failed.

Let our legislative committee get a move on, and let every regular physician work for its passage, co-operating with the State Association in its laudable and persistent efforts to protect the public from flagrant quackery, whatever may be one's individual opinion (like mine, for instance) as to what *ought* to be done. Let us do the best that *can* be done.

But, as to fraternizing with the pathies, that is a different proposition. If some of our members desire to serve on a mixed board, it does not follow, by any means, that we should invite all the other so-called "schools" to unite with us in our State Association. The State Association's journal (January) makes a strong plea for homologation—or miscegenation,—if I may use that word, with all kinds of pathies, and argues that they study the same text-books that we use in regular schools. That does not make them any less quacks from the standpoint of professional ethics; for the Principles of Medical Ethics—our *Magna Charta*—distinctly states (Chapter 11. Duties of Physicians): "It is inconsistent with the principles of medical science and it is incompatible with honorable standing in the profession for physicians to designate their practice as based on an exclusive dogma, *or a sectarian system of medicine.*"

Hence, so long as one claiming to be a physician holds on to the title "osteopath," "homeopath," or what-not, as a kind of trade mark to catch practice, he is not eligible to membership in the State Association of physicians, and I am sure the *State Journal of Medicine* does not voice the sentiment of the profession in making this plea for their recognition. If they will drop the title: call themselves simply "physicians," no one cares, and no one will ask what methods of practice they pursue, and I would be willing to receive all honorable men of whatever "school" into fellowship. But so long as they will not do this they can not be considered as eligible; in fact, their *sectarian title makes them quacks,* and we can not absorb the quacks.

